# Anti-aging interventions affect lifespan variability in sex, strain, diet and drug dependent fashion

**DOI:** 10.18632/aging.102037

**Published:** 2019-06-24

**Authors:** Andrzej Bartke, Tracy R. Evans, C.J.M. Musters

**Affiliations:** 1 Department of Internal Medicine, Geriatrics Research, Southern Illinois University School of Medicine, Springfield, IL 62794, USA; 2 Illinois State Museum Research and Collections Center, Springfield, Illinois 62703, USA; 3 Institute of Environmental Sciences, Leiden University, Leiden 2333 CC, The Netherlands

**Keywords:** longevity, variance, growth hormone, rapamycin, acarbose, 17 alpha estradiol, calorie restriction, mice

## Abstract

Decreased forkhead box O1 (FoxO1) activity induces hyperlipidemia and increased PPARγ, leading to hyperlipidemia in association with endoplasmic reticulum (ER) stress. In the liver, aging and comorbidities such as hyperlipidemia and diabetes significantly influence a wide variety of steatosis, but the underlying mechanisms are complex and remain elusive.

To establish the modulatory role of FoxO1 and the functional consequences of its altered interaction with PPARγ in the present study, we utilized a cell culture system, aged rats and diabetic db/db mice.

We found that, under ER stress, FoxO1 induces PPARγ-mediated lipid accumulation in aged rat livers. Our data showed that the FoxO1-induced hepatic lipid accumulation was negatively regulated by Akt signaling. PPARγ, a key lipogenesis transcription factor, was increased in aged liver, resulting in lipid accumulation via hepatic ER stress under hyperglycemic conditions. We further demonstrated that loss of FoxO1 causes a decline in PPARγ expression and reduces lipid accumulation. In addition, the interaction between FoxO1 and PPARγ was shown to induce hepatic steatosis in aging and db/db mice.

We provide evidence that, in aged rats, FoxO1 interaction with PPARγ promotes hepatic steatosis, due to hyperglycemia-induced ER stress, which causes an impairment in Akt signaling, such in aging-related diabetes.

## Introduction

In studies of aging, changes in the length of life are usually analyzed by comparing average (mean) or median longevity. Frequently, some estimate of maximal longevity is also considered. While values of the standard deviations or standard errors of the mean are routinely reported, the distribution of individual age at death is rarely analyzed or discussed. This contrasts with the recent interest in analyzing the distribution of biomarkers of aging using the statistical distance measure [1] to estimate the level of physiological dysregulation and to relate it to resilience and robustness during aging [2].

In a recent publication based on analysis of demographic data, Van Raalte and her colleagues reported that socio-economic status influences not only the mean longevity but also the variability of human life-span [3]. Using an example of Finnish women, these investigators showed that reduced mean longevity of less educated and less affluent people is associated with greater variability of life-span. The inverse relationship of life expectancy and life-span variation was seen also in other human populations [3]. The practical implication of these findings is that for both the individuals and the health care systems it is more difficult to predict the age at death of the less privileged people than of the more privileged strata or for the entire population.

Because of the potential significance of this relationship for the analysis of mortality data and physiological biomarkers in studies of aging as well as for various public health considerations, we thought that it would be of interest to determine whether interventions known to extend the average (or the average and the maximal) longevity of experimental animals have any effect on the variability of life-span. We hypothesized that extension of longevity by genetic, dietary or pharmacological means leads to reduction of life-span variability. However, inspection of data from the National Institute of Aging Interventions Testing Program (ITP) [4] and from our studies of the interactions of murine longevity genes with calorie restriction (CR) [5–7] indicated that reciprocal changes of longevity and its variability are not consistently observed. This suggested that our hypothesis would most likely need to be rejected and brought up a new question, namely, what factors influence variability of the lifespan. Here we report results of a study aimed at analyzing the effects of sex, strain, life-extending interventions and their interactions on life-span variation.

## RESULTS

**Figure 1:**
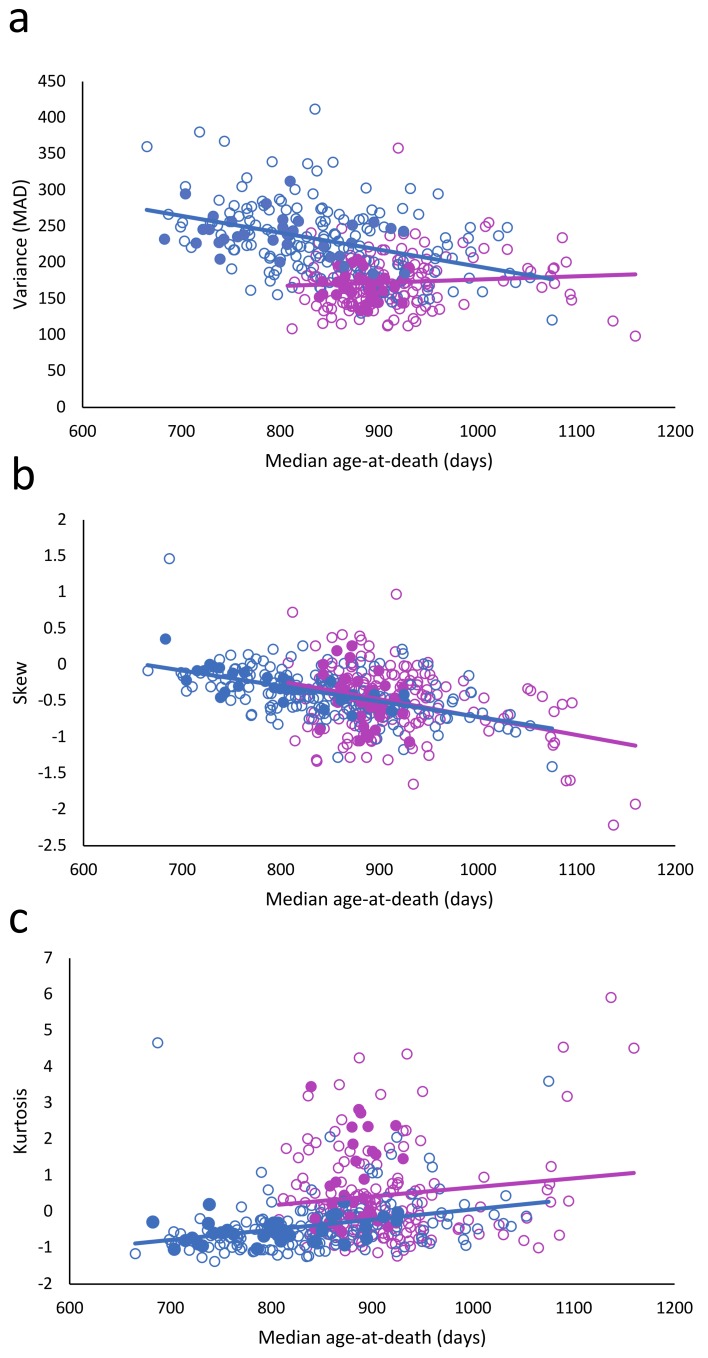
Relationship between longevity and variance (**a**), skewness (**b**), and kurtosis (**c**). Females: red circles; males: blue circles; filled circles: treated; and unfilled circles; untreated.

When the longevity of the mice in the ITP dataset was correlated to the variance in longevity, a strong decrease in variance with increasing longevity was observed. Skewness also decreased, while kurtosis, i.e., non-flatness, increased (Table 1). However, when gender was included in these relationships, we found that it strongly affected the results (Fig. 1; Table 2).

**Table 1:** Estimates of the regression coefficients of the fitted line of longevity against variance, skewness and kurtosis.

	Estimate	SE	t-value	df	𝛘^2^ (LRT)	p-value	
Variance (MAD)	-0.425	0.047	-9.125	1	71.57	<0.001	
Skewness	-0.442	0.047	-9.472	1	79.98	<0.001	
Kurtosis	0.325	0.049	6.681	1	42.19	<0.001	

In each test group, longevity is measured as the median age at death, variance as the Median Absolute Deviation (MAD) of age at death, skewness as the skewness of age at death, and kurtosis as the kurtosis of age at death. Estimate, standard error (SE) and t-value are from linear mixed models with research site as random effect variable. 𝛘^2^ and p-value are from a Likelihood Ratio Test (LRT) where the model with the independent variable is compared to the model without that variable.

**Table 2:** Estimates of contrasts of models of longevity against variance, skewness and kurtosis, including gender and the interaction between gender and independent variables.

		Estimate	SE	t-value	df	𝛘^2^ (LRT)	p-value		
Variance (MAD)	Intercept (F)	-0.623	0.067	-9.288					
	Gender (M)	1.035	0.089	11.659	1	115.83	<0.001		
	Median age	0.071	0.072	0.978	1	0.95	0.329		
	Interaction Gender x Median	-0.437	0.091	-4.782	1	20.47	<0.001		
Skewness	Intercept (F)	0.068	0.083	0.822					
	Gender (M)	-0.106	0.104	-1.024	1	1.03	0.310		
	Median age	-0.505	0.084	-5.986	1	33.74	<0.001		
	Interaction Gender x Median	0.064	0.107	0.591	1	0.35	0.556		
Kurtosis	Intercept (F)	0.299	0.077	3.887					
	Gender (M)	-0.579	0.105	-5.505	1	29.16	<0.001		
	Median age	0.182	0.085	2.129	1	4.51	0.034		
	Interaction Gender x Median	0.023	0.108	0.216	1	0.05	0.829		

Estimate, standard error and t-value are from linear mixed models with research site as random effect variable. 𝛘^2^ and p-value are from a Likelihood Ratio Test (LRT) where the model with the independent variable is compared to the model without that variable.

The most striking finding was that the variance of longevity is strongly sexually dimorphic (Fig. 1) (Table 2: 𝛘^2^ = 20.47; df = 1; p < 0.001). In females the variance did not decrease with the increased longevity (Table 2: 𝛘^2^ = 0.95; df = 1; p =0.329), while that in males did (𝛘^2^ = 20.47; df = 1; p < 0.001). The skewness decreased (𝛘^2^ = 33.74; df = 1; P < 0.001), equally in both female and males (𝛘^2^ = 0.35; df = 1; p = 0.556). Kurtosis increased slightly (𝛘^2^ = 4.51; df = 1; p = 0.034), but was significantly lower in males than females (𝛘^2^ = 29.16; df = 1; p < 0.001).

**Figure 2:**
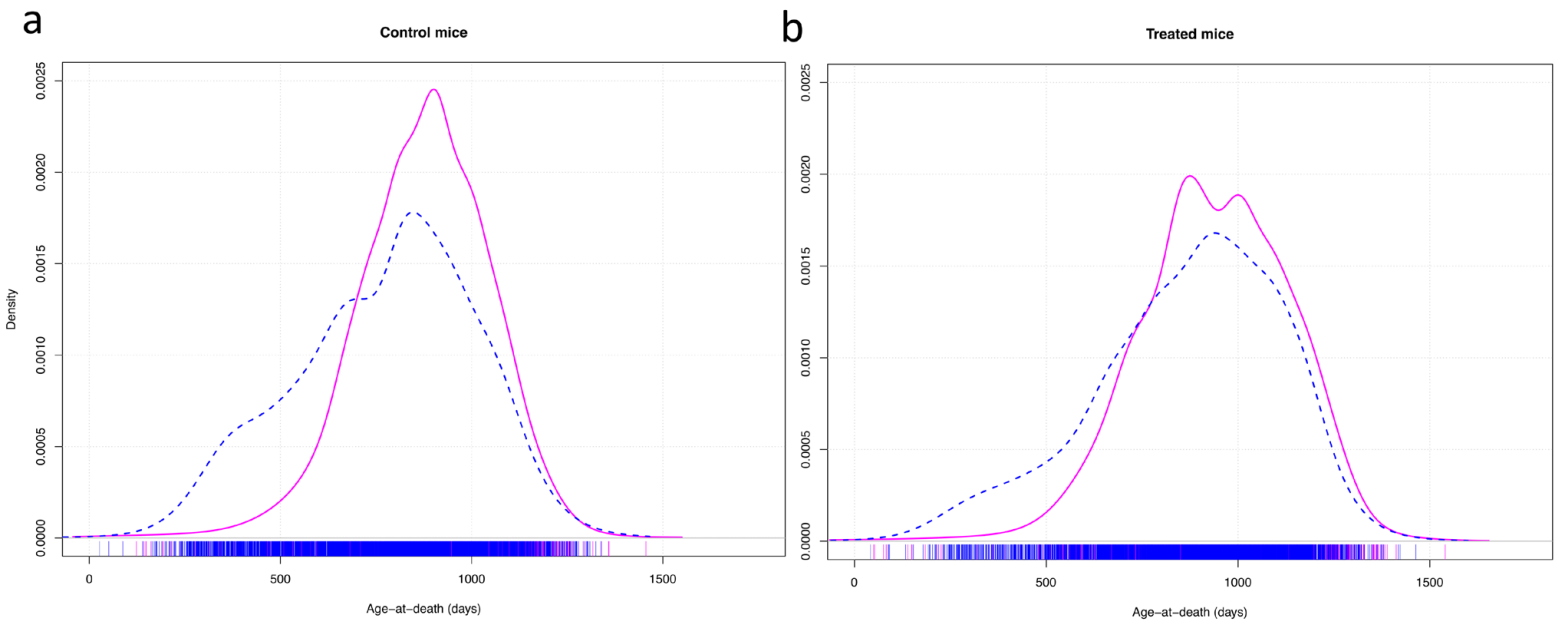
Density plots of the age at death of control mice (**a**) and mice treated with effective anti-aging pharmaceuticals (**b**). Cox-test for difference between females (solid red line) and males (dashed blue line) in survival with research site as random effect variable: control mice: z = 11.02, p < 0.001; treated mice: z = 6.95, p < 0.001.

A graphic representation of the variance in longevity of control mice vs mice treated with effective anti-aging compounds showed that, apart from the effect of the compounds on median longevity, they mainly seemed to decrease early death in males (Fig. 2).

**Figure 3:**
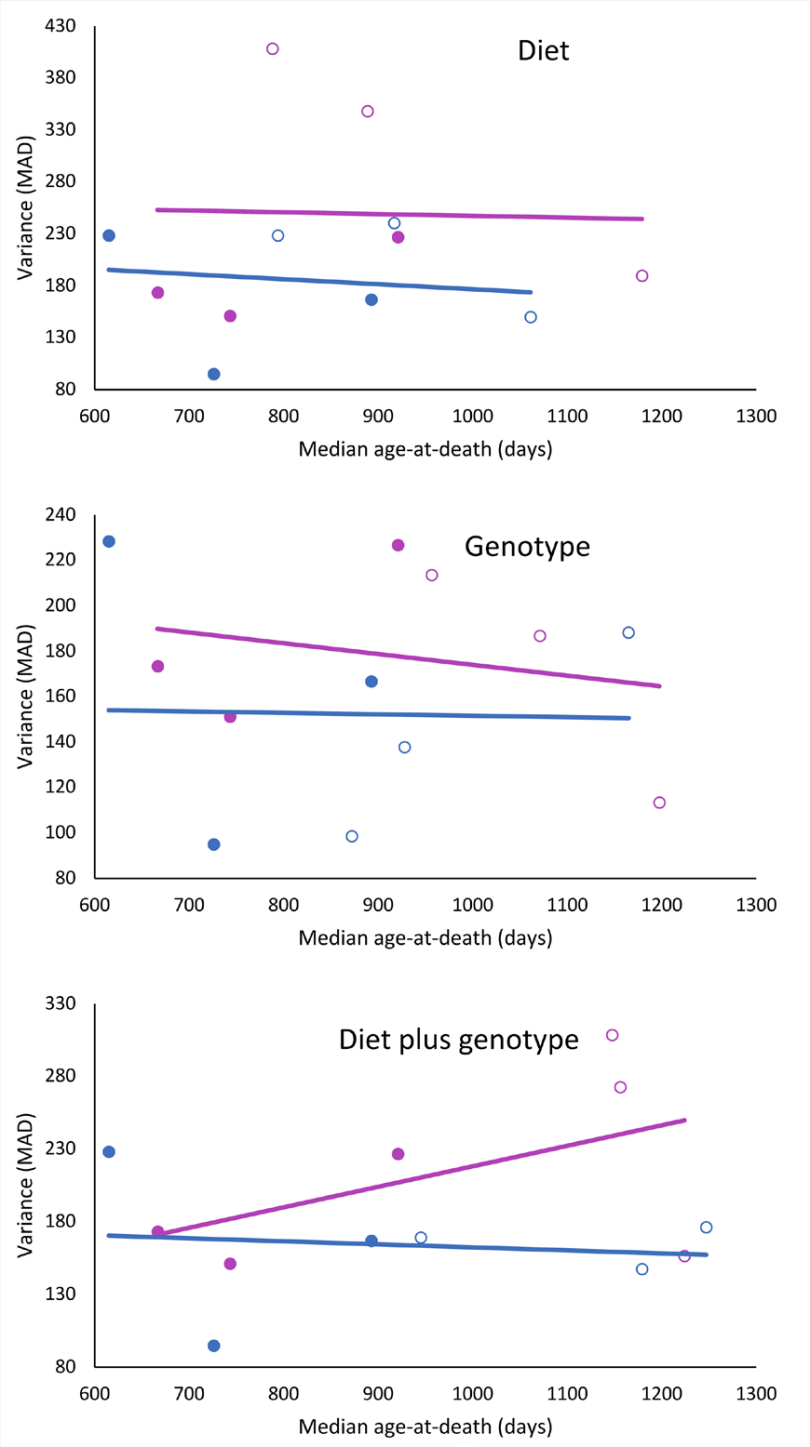
**Effects of CR, life extending mutations or both on the relationship to longevity and variance in female and male mice.** Females: red circles; males: blue circles; filled circles: treated; and unfilled circles; untreated.

The dataset of the longevity genes/CR studies showed no overall correlation between the median longevity per test group and the variance in longevity, measured as MAD per test group (𝛘^2^ = 0.25; df = 1; p = 0.617). When gender was taken into consideration and the studies were split according to effect of diet, genotype, or diet plus genotype, it was found that when mice had longevity extending genotypes and were treated with CR, variance of females increased near statistical significance (𝛘^2^ = 3.25; df = 1; p = 0.071), while in males this seemed not the case (Figure 3; Table 3).

**Table 3:** Estimates of contrasts of models of longevity with variance including gender and the interaction between gender and independent variables; CR, life extending mutations, or both.

		Estimate	SE	t-value	df	𝛘^2^ (LRT)	p-value	
Diet	Intercept (F)	0.375	0.442	0.850				
	Gender (M)	-0.756	0.625	-1.210	1	2.02	0.156	
	Median age	-0.032	0.433	-0.074	1	0.01	0.928	
	Interaction Gender x Median	-0.058	0.659	-0.088		0.01	0.914	
Genotype	Intercept (F)	0.298	0.494	0.603				
	Gender (M)	-0.576	0.616	-0.936	1	1.16	0.282	
	Median age	-0.192	0.457	-0.420	1	0.25	0.616	
	Interaction Gender x Median	0.124	0.644	0.192	1	0.07	0.798	
Diet+Gen.	Intercept (F)	0.379	0.450	0.843				
	Gender (M)	-0.810	0.454	-1.787	1	3.83	0.050	
	Median age	0.567	0.347	1.636	1	3.25	0.071	
	Interaction Gender x Median	-0.573	0.477	-1.202	1	1.97	0.161	

Estimate, standard error and t-value are from linear mixed models with strain as random effect variable. 𝛘^2^ and p-value are from a Likelihood Ratio Test (LRT) where the model with the independent variable is compared to the model without that variable.

**Figure 4:**
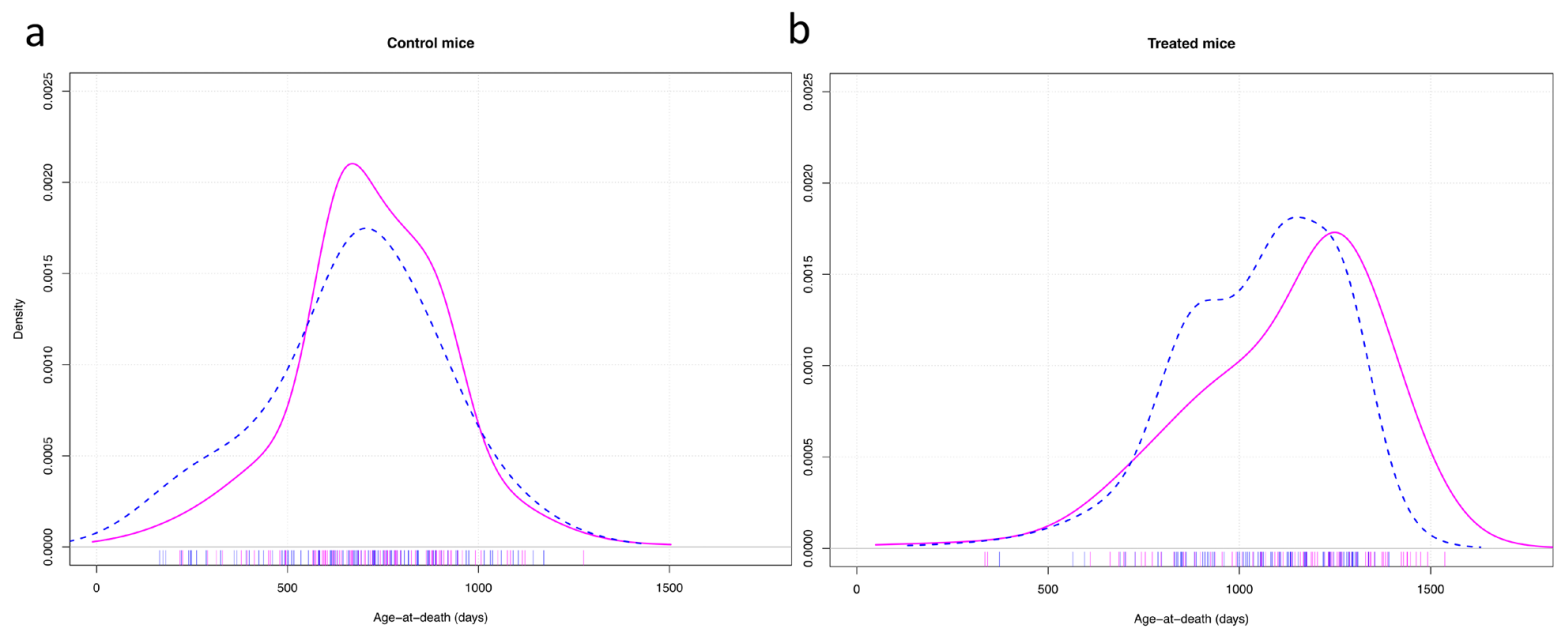
Density plots of the age of death of control mice (**a**) and mice treated with anti-aging diet as well as having anti-aging genotypes (**b**). Cox-test for difference between females (solid red) and males (dashed blue) in survival with strain as random effect variable: control mice: z = 0.57, p = 0.57; treated mice: z = 3.97, p < 0.001.

The graphic representation of the variance in longevity of control mice vs long-lived mutant mice treated with CR showed that, apart from the life extending effect of the treatment on median longevity, the gender difference in longevity was due predominantly to some males dying relatively early (Fig. 4).

## DISCUSSION

Results of the present study indicate that interventions which extend longevity in laboratory mice can also alter the distribution and the variance of age at death. The presence, the magnitude, and apparently also the direction of these changes depend on the sex of the animals and the nature of the interventions. In the large data set from the ITP studies, increased longevity was associated with reduced variance in males (p<0.001) but not in females. Moreover, distribution of the data suggested a possible trend for variance in female data changing in the opposite direction (Fig 1). In long-lived mice with GH-related mutations, subjected to CR, variance was not related to longevity in males, but seemed to increase with longevity in females (p=0.071).

Sexual dimorphism in the responses to longevity-extending interventions is not unexpected and our findings show that the relationship of the distribution of individual age at death data to the average life span of the population is also sex-dependent. Thus, the present results add to the growing body of evidence that various aspects of aging, including longevity, as well as responses to various anti-aging interventions cannot be predicted from data obtained in individuals of different sex [8].

The reciprocal relationship of human longevity and its variablitiy that Van Raalte, *et al.* [3] found in various data sets were from segments of the same population separated on the basis of income, educational attainment, and/or type of employment. The biological basis of the well-documented impact of socio-economic factors on human aging is believed to include differences in diet, health-related behaviors, and access to as well as quality of health care, although significance of the latter factors in Finland and other countries with excellent health care systems is presumably small or absent.

Since animals in each of the four examined cohorts were genetically heterogeneous and three of the four cohorts were relatively small, this study does not allow any firm conclusion on the possible differences between strains. Studies including large genetically distinct populations, different inbred strains and preferably also their crosses would be needed to address this issue.

What can be concluded from our analysis is that in studies of anti-aging interventions in laboratory animals the changes in variability of lifespan cannot be predicted from changes in longevity. It is interesting to speculate that this may be related to differences in the mechanisms responsible for lifespan extension by the various interventions.

In the data sets we analyzed, there were examples of differences in the proportion of early deaths in females and males as well as in long-lived (mutant, calorie restricted or drug-treated) vs control (wild type, fed ad libitum, or untreated) animals and the slopes of survival curves also differed in some cases. Skewness and kurtosis were as expected. The differences in the distribution of individual lifespans may have contributed to the observed differences in variation. For example, decrease in the occurrence of early deaths likely contributed to longevity of drug-treated males in the ITP studies [9]. This and other examples of the role of early deaths in determining the longevity of the whole cohort resemble demographic findings on the effects of socio-economic factors in human populations [3].

Analysis of distribution of individual age at death values suggests a differential impact of the examined anti-aging interventions at different segments of life history. This, in turn, is likely related to different mechanisms of their actions. Significant correlation of extended longevity with reduced variance of individual death in various human populations [3] and in male mice from the ITP cohort (Fig 1), may have been due, in part, to the “ceiling effect” as longevity approaches the value of maximal lifespan for the species. More likely, it reflects greater proportion of early deaths of various etiologies in groups with shorter median lifespan. Intriguingly, trajectories of several physiological processes mechanistically related to aging are more variable in short-lived than in exceptionally long-lived people [10].

Regardless of the mechanisms involved, our data suggest that analysis of changes in the variability of life-spans in response to various interventions can provide additional information on the nature of the observed effects.

## MATERIALS AND METHODS

We used data on the length of life of individual mice (age at death measured in days) from the ITP [4] and from studies conducted at Southern Illinois University (SIU) [5–7]. In the ITP, genetically heterogeneous (UM-HET3) female and male mice derived from crosses of four inbred strains were treated with various compounds starting in early adulthood and continuing until they died or were euthanized when judged to be moribund. These studies were conducted in parallel at three collaborating institutions. Both significant extension of longevity and absence of effects were reported in peer-reviewed literature [9,11–15]. A listing of the compounds, their actions and the percent increase in median longevity of treated animals are listed in Table 4 along with references to publications reporting longevity data.

**Table 4:** Percent increase in median lifespan due to pharmacological agents administered in the Interventions Testing Program.

Drug	Effects, mode of action	Δ Median lifespan		Citation
		Females	Males	
Acarbose	Inhibitor of intestinal alpha-glocosidase	5	22	Harrison *et al.,* 2014
Aspirin	anti-inflammatory, antithrombotic and antioxidant	ns	8	Stong *et al.,* 2008
17 alpha-estradiol	neuroprotection	ns	12	Harrison *et al.,* 2014
Dietary glycine	anti-inflammatory	4	1	Brind *et al.,* 2018
NDGA	Antioxidant, anti-inflammatory	ns	8-10	Harrison *et al.,* 2014
Protandim (mixture of plant extracts)	Nrf activation; increase of antioxidant defenses	ns	7	Strong *et al.,* 2016
Rapamycin	Inhibitor of mTORC1	18	10	Miller *et al.,* 2011

Footnote: For some of the above listed drugs, additional studies were conducted using different doses and or starting treatment at a different age which resulted in somewhat different % extension of median life-span.

The effects of pharmacological anti-aging interventions in the ITP studies were analyzed using the data from treated mice with documented life extension and matching controls. These groups include animals treated with acarbose [9], aspirin [11], 17-α-estradiol [9], dietary glycine [15], nordihydroguaiaretic acid [9,11], Protandim^TM^ (mixture of plant extracts) [14], or rapamycin [16]. Longevity variance was examined in relation to treatment and sex while taking into account differences in testing laboratories [4].

A second dataset was created with individual longevity data of mice from three separate experiments on the interaction of calorie restriction (CR) with one of three life-extending mutations [5–7]. These experiments used mice with suppression of growth hormone (GH) signaling by a mutation interfering with differentiation of three types of anterior pituitary cells leading to hypopituitarism including GH deficiency [17,18], deletion of GH releasing hormone (*GHRH*) gene leading to isolated GH deficiency [19], or deletion of the GH receptor (*GHR*) gene leading to GH resistance [20]. Approximately half of the animals in each genotype/sex group were fed ad libitum (AL), while the remainder were subjected to calorie restriction (CR) starting in early adulthood by feeding them 60 -70% of the amount of food consumed during the preceding week by age, sex, and genotype matched AL mice. The employed mutations were maintained on a heterogeneous genetic background which was different for each mutation and different from the genetic make-up of the ITP mice. Each of the three mutations used in these studies increases longevity of both females and males while the effects of CR on longevity were mutation- and sex-dependent [5–7]. Percent increase of median longevity in each genotype/sex/diet group is listed in Table 5.

**Table 5:** Interactive effects of calorie restriction and genetic suppression of growth hormone signaling on median longevity in each genotype/sex/diet group.

Strain	Diet	Female	Male	Action	Citation
Ames N	CR	20	26	Hypopituitarism including GH deficiency	Bartke *et al.,* 2001
Ames dwarf	AL	44	20		
Ames dwarf	CR	65	72		
*GHR N*	CR	28	19	deletion of the GH receptor (GHR) gene leading to GH resistance	Bonkowski *et al.,* 2006
*GHR KO*	AL	30	31		
*GHR KO*	CR	25	32		
*GHRH N*	CR	18	29	deletion of GH releasing hormone (GHRH) gene leading to isolated GH deficiency	Sun *et al.,* 2013
*GHRH KO*	AL	44	51		
*GHRH KO*	CR	74	54		

There are 24 groups: three strains (Ames, GHR, GHRH); two sexes; two diets (CR and AL); and two genotypes (Normal and dwarf or knock-out). Normal (N) indicates normal siblings of mutant mice.

Longevity variance was examined in relation to different groups (genotype, diet, and sex combinations). Interventions that were ineffective (such as CR in *GHR -/-* mice) were included to account for the possibility that CR could alter variance without altering the median.

We performed all statistical analysis using R software 3.4.4 [21]. For studying the relationship between longevity and variance in longevity we calculated the median age at death of the females and males of all available test groups. We chose median instead of mean because we could not assume that the variance in longevity was normally distributed. For the variance in longevity we calculated the MAD, i.e., the Median Absolute Deviation, a robust metric fit for non-normal distributions [22]. Median, MAD, skewness, and kurtosis were all calculated with the function *describeBy()* of the package *psych* [23]. For testing the relationships between longevity and variance, skewness and kurtosis, the function *lmer()* of the package *lme4* [24] for linear mixed-effect models were applied, combined with the *drop1()* function for the Likelihood Ratio Test (LRT). For testing the difference in survival between females and males, the Cox test for mixed effects was used, i.e., the function *foxme()* of the package *foxme* [25]. As random effect variable, the research site was included in all analyses of the ITP data and the strain in all analyses of the SIU (mutations/CR) data. The graphs were constructed using *scatterplot()* and *densityPlot()*, both of the package *car* [26]. We preferred density plots over Kaplan-Meier survival plots, because they show more clearly where the differences between females and males occurred.
